# NFAT5 directs hyperosmotic stress-induced fibrin deposition and macrophage infiltration via PAI-1 in endothelium

**DOI:** 10.18632/aging.202330

**Published:** 2020-12-19

**Authors:** Pingping Ma, Guang Li, Xiaorui Jiang, Xinkun Shen, Hong Li, Li Yang, Wanqian Liu

**Affiliations:** 1 Key Laboratory of Biorheological Science and Technology, Ministry of Education, Bioengineering College, Chongqing University, Chongqing 400044, China; 2 Chongqing University Cancer Hospital, Department of Radiotherapy, Chongqing University, Chongqing 400044, China; 3 School and Hospital of Stomatology, Wenzhou Medical University, Wenzhou 325027, China; 4 Obstetrics and Gynecology Department, Xinqiao Hospital, Army Medical University, Chongqing 400037, China

**Keywords:** high salt, atherosclerosis, endothelial cells, NFAT5, PAI-1

## Abstract

Although stress can significantly promote atherosclerosis, the underlying mechanisms are still not completely understood. Here we successfully unveiled that high salt-induced nuclear factor of activated T cells 5 (NFAT5) control the endothelial-dependent fibrinolytic activity and the inflammatory adhesion-related molecules expression through regulation of plasminogen activator inhibitor-1 (PAI-1). We first observed that high salt diets instigated the expression of NFAT5 and PAI-1 in the endothelium which brought about the fibrin deposition and macrophage infiltration in the atherosclerotic arteries of ApoE^-/-^ mice. Overexpression of NFAT5 increased PAI-1-mediated antifibrinolytic activity and activated inflammatory adhesion-related genes in endothelial cells. Knockdown of NFAT5 by siRNA inhibited the expression of PAI-1, antifibrinolytic and adhesive molecules. Moreover, chromatin immunoprecipitation assay demonstrated that high salt intake significantly promoted the binding of NFAT5 to PAI-1 promoter (TGGAATTATTT) in endothelial cells. Our study identified that NFAT5 has great potential to activate the PAI-1-mediated fibrinolytic dysfunction and inflammatory cell adhesion, thus promoting high salt-induced atherosclerosis disease.

## INTRODUCTION

Atherosclerosis (AS) is a major global disease, which is initiated and exacerbated by the chronic dysfunction of vascular endothelium lining along the blood vessels [1, 2]. Epidemiological studies have reported that the development of AS is heavily influenced by cardiovascular risk factors (e.g., high-salt intake, dehydration, and diabetes) [3–5]. In the course of atherosclerosis, endothelial cells (ECs) detect and translate cardiovascular risk factors into injurious responses, resulting in subsequent fibrin deposition, macrophage infiltration, foam cell formation, as well as other pathological changes [6–8]. Accumulating data showed that high salt (HS) intake brings about the inflammation and the over-expression of inflammatory adhesive molecules, and coagulation mediators (e.g., von Willebrand factor, vWF) in ECs [3, 9]. Although extensive studies have revealed that high-salt intake is associated with the dysfunction of endothelium, the key molecular events linking hypertonic stress to atherogenic responses remain undetermined.

Plasminogen activator inhibitor-1 (PAI-1), a physiological plasmin activator inhibitor, will be activated in ECs during AS and other cardiovascular events [10, 11]. Through inhibiting the activation of urokinase plasminogen activator (PLAU) and tissue plasminogen activator (PLAT), PAI-1 reduces the formation of active plasmin in blood circulation and increases the deposition of fibrin in tissues (e.g., liver, heart, and kidney), respectively [12, 13]. PAI-1 is also observed in several non-fibrinolytic processes (e.g., cell adhesion, migration, and infiltration) [14–16]. For instance, elevated PAI-1 can significantly increase leukocytes infiltration and induce tissue injury. In the context of AS, the local high expression of PAI-1 has been determined as a key risk factor for atherogenic events. Previous studies claimed that the expression of PAI-1 in HS intake may give rise to renal fibrosis and cardiovascular injury [17, 18]. However, the mechanism of high-salt-induced endothelial PAI-1 expression and its function in AS remained mostly unclear.

Nuclear factor of activated T cells 5 (NFAT5) is a common transcription factor that regulates the responses of cells to osmotic stress [19]. The activated NFAT5 can further promote the expression of its target genes essential for angiogenesis [20], arterial stiffening [21], as well as tumor metastasis [22]. Increased NFAT5 activity drives the formation of lesions by promoting macrophage survival/migration as well as coagulation [23, 24, 9]. Whereas the deficiency of NFAT5 will result in suppressing the infiltration of chronic macrophage and the formation of AS. In addition, the expression of inflammatory genes and adhesion molecules under hypertonic stress condition can also be effectively regulated by NFAT5 [25].

In the present study, we revealed a new mechanism that HS significantly activates PAI-1 via NFAT5 to further promote fibrin deposition and macrophage infiltration in ECs. The impact of HS diet on AS formation, fibrin deposition, and macrophages infiltration in the artery of ApoE^−/−^ mice was first evaluated. Then, the effects of HS on the expression of PAI-1, NFAT5, and downstream target gene in ECs were investigated. The molecular mechanism of NFAT5 activating downstream signal pathways via PAI-1 was further studied. All results indicated that NFAT5 was the key regulator of PAI-1-mediated fibrin deposition and macrophages infiltration.

## RESULTS

### HS intake predisposes fibrin deposition, macrophage infiltration and AS in aortas of ApoE^-/-^ mice

**Figure 1:**
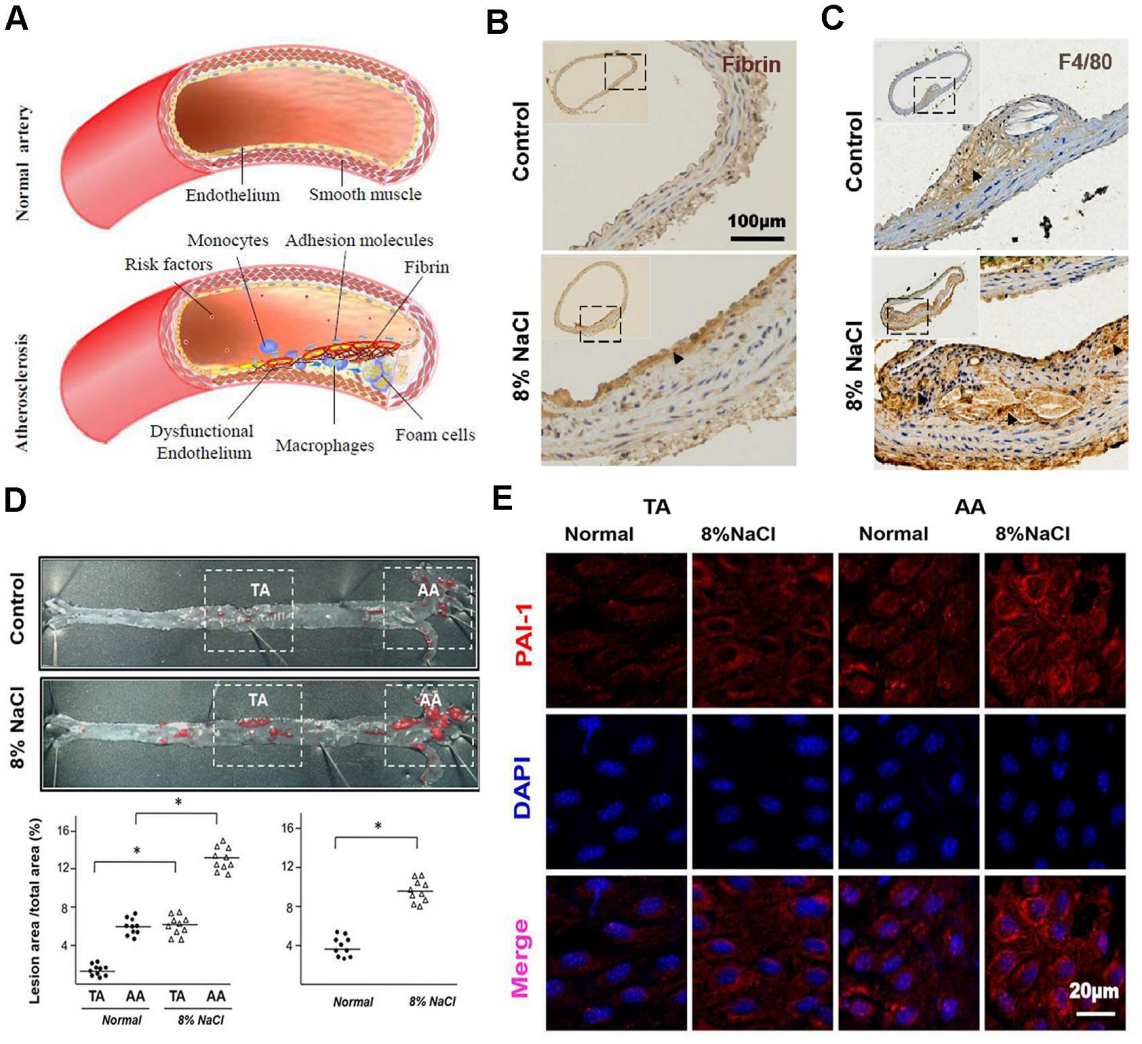
**High-salt intake predisposes fibrin deposition, macrophage infiltration and atherosclerosis formation in aortas of ApoE^-/-^ mice.** (**A**) The schematic diagram shows the process of fibrin deposition, macrophage infiltration and atherosclerosis formation. (**B**, **C**) Representative sections of the AA region in ApoE^-/-^ mice stained for fibrin and macrophage marker F4/80 (n = 7). Nuclei, hematoxylin staining. Fibrin and F4/80-positive macrophages were marked respectively by arrow heads. (**D**) Oil Red O staining of the artery and quantification of percentage lesion areas in the thoracic aorta (TA) and aortic arch (AA) of ApoE^-/-^ mice (n = 10) fed with a normal or high-salt diet for 12 weeks. (**E**) En face immunofluorescent staining of PAI-1 (red) in ECs of TA and AA regions of ApoE^-/-^ mice in normal and high salt groups after 4 weeks feeding. Nuclei were stained by DAPI. All data were presented as mean ± SEM, N≥7. *p < 0.05 versus control group.

Fibrin deposition, macrophages infiltration as well as liquid accumulation induced by cardiovascular risk factors in the arterial wall have been implicated in the development of AS (Figure 1A). To determine the effects of HS intake on AS, we first verified the fibrin deposition and macrophages infiltration at the site of atherosclerotic lesions in ApoE^-/-^ mice by staining fibrin and F4/80 (macrophage marker). The results of immunohistochemistry staining (Figure 1B) displayed that HS intake significantly enhanced the fibrin deposition in the subendothelial layer of the aortic artery in ApoE^-/-^ mice. Compared with the 0.8% group, ApoE^-/-^ mice in HS group also had a significant increase in the number of macrophages invading into the aortic plaque (Figure 1C). According to Supplementary Figure 1, it was found that the serum sodium and cholesterol levels (~ 149.5 mmol/L and ~ 4.4 mmol/L) in the high salt group were significantly higher than those (~ 155.4 mmol/L and ~ 7.2 mmol/L) in the control group. Next, we detected the formation of aortic plaques in ApoE^-/-^ mice after feeding for 12 weeks with normal (0.8% NaCl) or HS (8% NaCl) diet. The results of Oil Red O staining (Figure 1D) further show that HS significantly promoted atherosclerotic lesions, especially in the aortic arch (AA) region, indicating that HS intake predisposes to AS. This suggested that HS intake activates fibrin deposition and macrophage infiltration during AS.

### HS increases the expression of PAI-1 in ECs

Other studies have reported the association of PAI-1 with endothelial fibrinolysis and leukocyte infiltration [13, 15, 26]. Therefore, we evaluated the expression of PAI-1 in ECs from different parts (renal cortex, outer and inner renal medullae) of the kidney. The osmotic pressure of these different parts had been proved to increase gradually under physiological conditions. The results of immunohistochemistry staining (Supplementary Figure 2A) displayed that, compared to the renal cortex, the PAI-1 expression was higher in the other two hyperosmolar regions (outer and inner renal medullae), indicating that the expression of PAI-1 was related to the osmotic pressure of ECs (marked with CD31).

An *in vivo* study was further done to investigate HS diet and its association to an elevated PAI-1 expression in the arterial ECs. RT-PCR results (Supplementary Figure 2B) showed that the gene expression of PAI-1 in HS group was significantly higher than that in the control group. The results of En face immunofluorescent staining (Figure 1E) also displayed that the expression of PAI-1 was up-regulated in the AA of HS group compared with that of the control group.

### HS intake induces endothelial fibrinolytic dysfunction and thrombi

**Figure 2:**
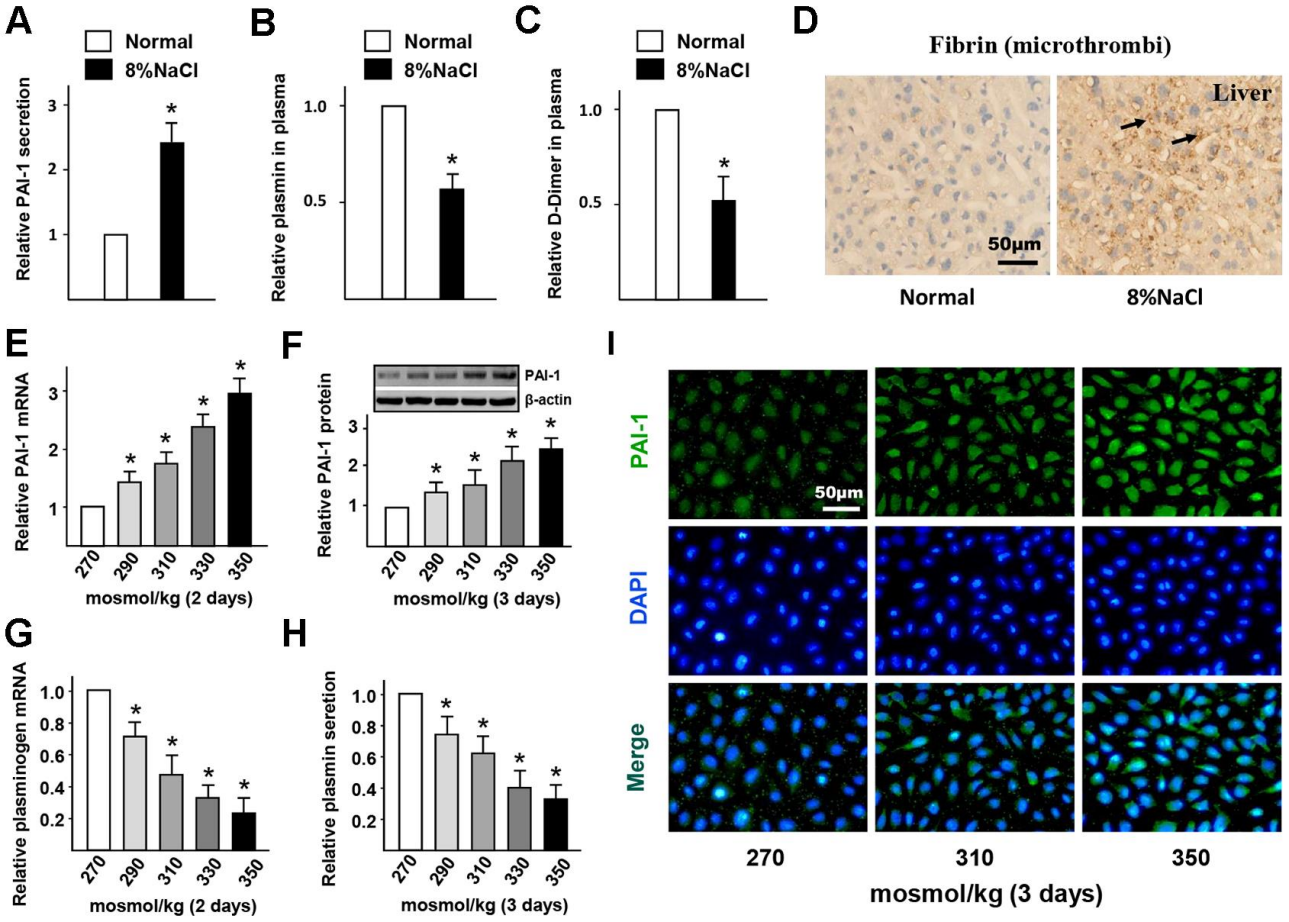
**High-salt intake induces endothelial fibrinolytic dysfunction and thrombi.** (**A**–**C**) Protein levels of PAI-1, active plasmin and D-Dimer in plasma of mice in normal and high salt groups after 4 weeks feeding. (**D**) Representative sections of the livers in ApoE^-/-^ mice stained for fibrin. Nuclei, hematoxylin staining. Microthrombi were marked by arrowheads. (**E**, **F**) mRNA and protein expression of PAI-1 in HUVECs that cultured with different hyper-osmotic media (270, 290, 310, 330 and 350 mosmol/kg) for two or three days. 270 mosmol/kg was as the control. (**G**, **H**) mRNA and protein level of plasmin in HUVECs that cultured with different hyper-osmotic media for two or three days. 270 mosmol/kg was as the control. (**I**) Representative immunofluorescent staining of PAI-1 (green) in HUVECs that exposed to different hyper-osmotic media for three days. Nuclei were stained by DAPI. All data were presented as mean ± SEM, N≥3. *p < 0.05 versus control group.

Given that endothelial PAI-1 acts as a critical mediator of blood fibrinolysis, we hypothesized that high-salt-induced PAI-1 from ECs *in vivo* might attenuate blood fibrinolysis and increase thrombosis. Elevated PAI-1 blocks plasminogen to convert the active plasmin by inhibiting the activity of PLAT and PLAU, which in turn impairs fibrin degradation and promotes the formation of cross-linked fibrin polymers. The levels of PAI-1, plasmin, and D-dimer (a common indicator of coagulation function) in mice plasma, and the number of microthrombi in mice liver (thrombosis susceptible tissue) were further evaluated to determine the effects of HS intake on blood fibrinolysis. From the ELISA results (Figure 2A–2C), it was found that the level of PAI-1 in the HS group was significantly higher than that in the control group, while the levels of plasmin and D-dimer were significantly lower. The immunohistochemistry staining results (Figure 2D) further proved that there was an obvious increase in the number of microthrombi within the capillaries of the liver of high-salt intake groups. These results suggested that HS diet could elevate the PAI-1 expression in AS which in turn impairs the fibrinolytic function of ECs.

To further verify the promotion of HS on endothelial PAI-1 expression and fibrinolytic dysfunction, some media with different osmolarities (270 - 350 mosmol/kg) were used to culture HUVECs. The results of RT-PCR and western blot (Figure 2E, F) displayed that the gene and protein expression of PAI-1 was significantly up-regulated in a dose-dependent manner. Immunofluorescent staining results (Figure 2I) also proved that HS promoted the PAI-1 expression in HUVECs. Correspondingly, the mRNA expression of plasminogen in HUVECs and the levels of active plasmin in the supernatant were significantly decreased under HS conditions (Figure 2G, 2H). This confirmed that HS induces PAI-1 expression and impairs fibrinolytic function in ECs.

### HS promotes adhesion molecules expression and inflammatory cells infiltration in ECs via PAI-1

**Figure 3:**
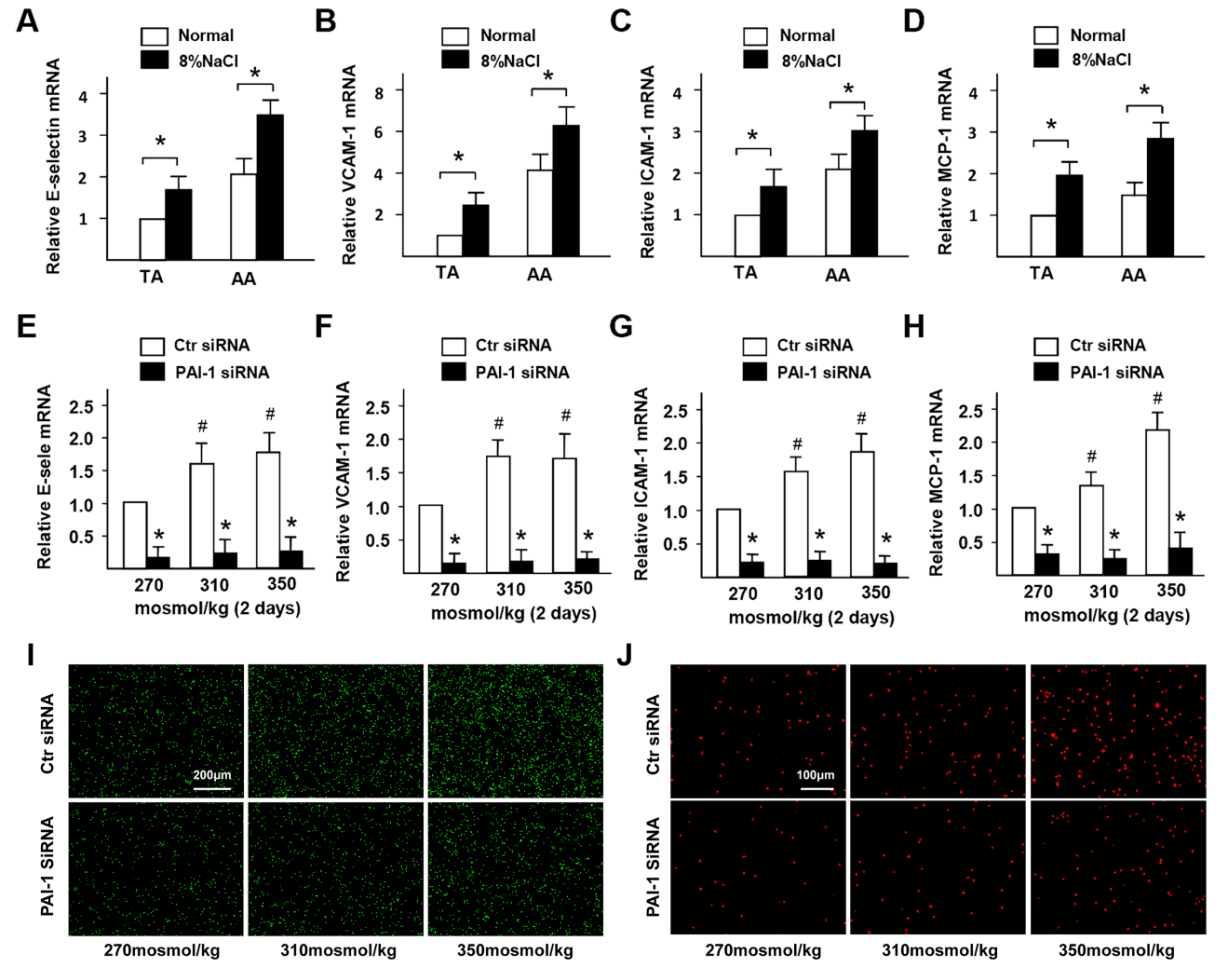
**High-salt promotes adhesion molecules expression and inflammatory cells infiltration in ECs via PAI-1.** (**A**–**D**) mRNA expression of adhesion molecules (E-selectin, VCAM-1, ICAM-1, and MCP-1) in TA and AA regions of ApoE^-/-^ mice in normal and high salt groups after 4 weeks feeding. (**E**–**H**) mRNA expression of adhesion molecules (E-selectin, VCAM-1, ICAM-1, and MCP-1) in HUVECs that were treated with Ctr siRNA or PAI-1 siRNA under high-salt condition for two days. (**I**) Representative images of adherent monocytes to HUVECs that were treated with Ctr siRNA or PAI-1 siRNA under high-salt condition. (**J**) Representative images of infiltrated monocytes into HUVECs that were treated with Ctr siRNA or PAI-1 siRNA under high-salt condition. All data were presented as mean ± SEM, N≥3. *p < 0.05 versus control group.

Previous studies demonstrated that PAI-1 contributes to neutrophil infiltration. To confirm whether the elevated PAI-1 also contributes to macrophages infiltration in the artery wall of mice with HS diet, the expression of adhesion molecules in endothelial cells was measured. The results of RT-PCR (Figure 3A–3D) revealed that the E-selectin, VCAM-1, ICAM-1, and MCP-1 genes were significantly activated in the HS diet group. It was further confirmed that PAI-1 siRNA could significantly inhibit this HS-induced up-regulation in HUVECs (Figure 3E–3H). In addition, we observed that there was less adhesion and infiltration of monocytes under HS condition when PAI-1 was down-regulated by siRNA according to cell adhesion and transwell migration results (Figure 3I, 3J). In summary, these results indicated that HS-promoted PAI-1 contributes to the dysfunction of inflammatory cell adhesion and infiltration to ECs.

### HS promotes NFAT5 nuclear translocation in ECs

**Figure 4:**
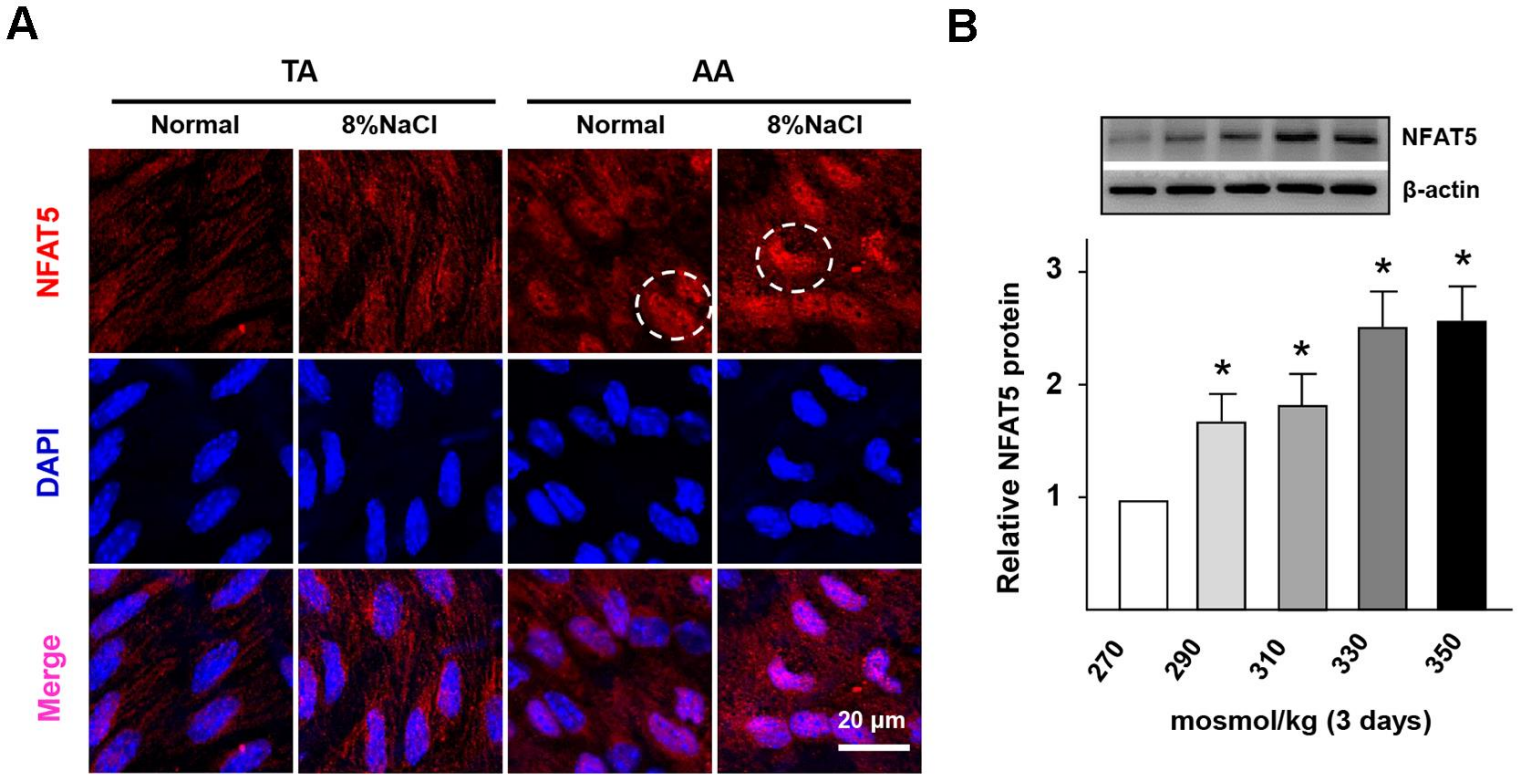
**High-salt intake induces NFAT5 nuclear translocation in ECs.** (**A**) En face immunofluorescent staining of NFAT5 (red) in ECs of TA and AA regions of ApoE^-/-^ mice in normal and high salt groups after 4 weeks feeding. Nuclei were stained by DAPI. NFAT5 nuclear translocation in ECs was marked by circles. (**B**) Protein expression of NFAT5 in HUVECs that exposed to different hyper-osmotic media for three days. All data were presented as mean ± SEM, N≥3. *p < 0.05 versus control group.

It has been claimed that NFAT5 could be significantly activated under intracellular hyperosmotic pressure [27]. Thus, we further investigated the difference of NFAT5 expression in the artery wall between normal and HS groups. From the RT-PCR results (Supplementary Figure 3A), it was found that the gene expression of NFAT5 in HS group was significantly higher than that of the control group. The results of En face immunofluorescent staining (Figure 4A) also presented that HS diet significantly increased the protein expression of endothelial NFAT5 in the AA region. Moreover, Figure 4A showed that NFAT5 expressed more in the cytoplasm of the control group, but gradually translocated into the endothelial nucleus under HS condition.

Next, with the increase of NaCl concentration, the gene and protein expression of NFAT5 was promoted gradually in HUVECs (Supplementary Figure 3B, Figure 4B). In addition, the gene expression of vWF (NFAT5 target gene) was also significantly up-regulated under HS conditions (Supplementary Figure 3C). Thus, these results demonstrated that the HS treatment could significantly increase the expression and nuclear translocation of NFAT5 in ECs.

### HS induces the dysfunction of PAI-1-dependent fibrinolysis and macrophages infiltration in ECs via NFAT5

**Figure 5:**
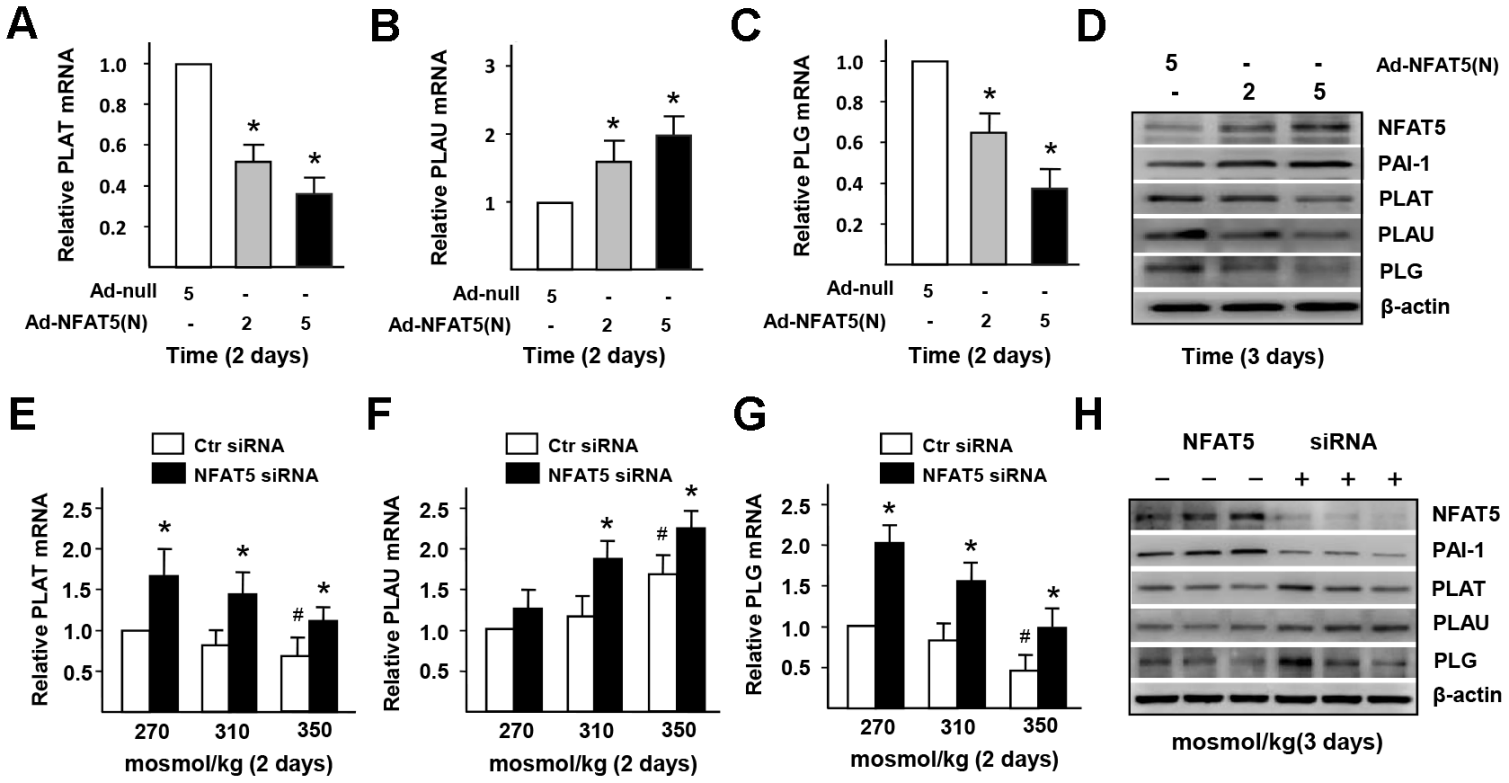
**High-salt induces the dysfunction of PAI-1-dependent fibrinolysis in ECs via NFAT5.** (**A**–**D**) mRNA and protein expression of fibrinolysis genes (PLAT, PLAU and PLG) in HUVECs that treated by Adenovirus-null (Ad-null) or Adenovirus-NFAT5 (Ad-NFAT5). (**E**–**H**) mRNA and protein expression of fibrinolysis genes (PLAT, PLAU and PLG) in HUVECs that transfected with Ctr siRNA or NFAT5 siRNA under high-salt condition. All data were presented as mean ± SEM, N≥3. *p < 0.05 versus control group.

In order to investigate whether NFAT5 regulates HS-inducted the dysfunction of fibrinolysis in ECs, we overexpressed the expression of NFAT5 using Ad-NFAT5 in HUVECs. The results of RT-PCR and western blot (Figure 5A–5D). displayed that the gene and protein expressions of PLG and PLAT were down-regulated after treating with Ad-NFAT5. However, overexpression of NTAT5 increased the mRNA expression of PLAU but decreased its protein expression. Contrarily, we suppressed the NFAT5 expression in HUVECs with siRNA. The results of RT-PCR and western blot (Figure 5E–5H) demonstrated that the gene and protein expressions of PLG, PLAT, and PLAU in NFAT5 siRNA-treated group were significantly higher than that in the control group. ELISA and statistical analysis (Supplementary Figure 4A) further showed that the concentration of HS-inhibited plasmin was elevated in the NFAT5 siRNA-treated group. These results suggested that NFAT5 mediated the expression and convert of plasminogen to active plasmin.

**Figure 6:**
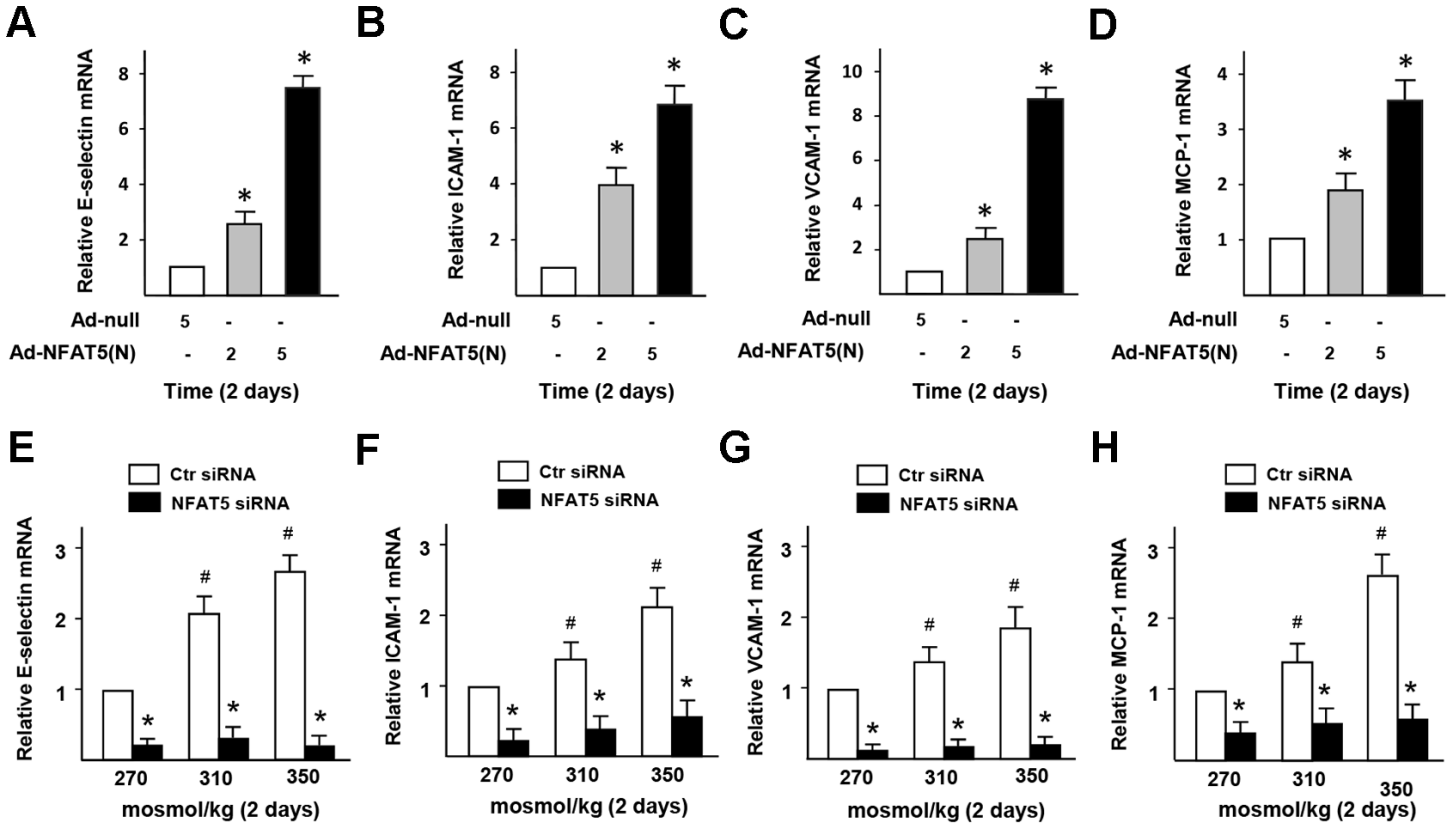
**High-salt induces the dysfunction of monocytes adhesion and infiltration in ECs via NFAT5.** (**A**–**D**) mRNA expression of adhesive molecules (E-selectin, ICAM-1, VCAM-1, and MCP-1) in HUVECs that treated by Ad-null or Ad-NFAT5. (**E**–**H**) mRNA expression of adhesive molecules (E-selectin, ICAM-1, VCAM-1, and MCP-1) in HUVECs that transfected by Ctr siRNA or NFAT5 siRNA under high-salt condition. All data were presented as mean ± SEM, N≥3. *p < 0.05 versus control group.

To investigate whether HS-inducted the dysfunction of monocytes adhesion in ECs is related to NFAT5, we checked the expression of adhesive molecules in HUVECs that treated by Ad-NFAT5. The results of RT-PCR (Figure 6A–6D) displayed that the gene expression of E-selectin, VCAM-1, ICAM-1, and MCP-1 in Ad-NFAT5 group was significantly higher than that in the control group. In the NFAT5-overexpressed cells, PAI-1 knockdown significantly down-regulated adhesion-related genes (E-selectin, VCAM-1, ICAM-1, and MCP-1) and increased PLAT in NFAT5-overexpressed cells, but had no effect on the expression of PLAU and PLG genes (Supplementary Figure 5A–5G). In addition, knockdown of NFAT5 with siRNA in HS-treated ECs also significantly inhibited the gene expression of E-selectin, VCAM-1, ICAM-1, and MCP-1 (Figure 6E–6H). These results suggested that the elevated PAI-1 through HS-induced NFAT5 promoted the dysfunction of endothelial fibrinolysis and monocytes adhesion.

### NFAT5 directly regulates PAI-1 transcription in ECs

**Figure 7:**
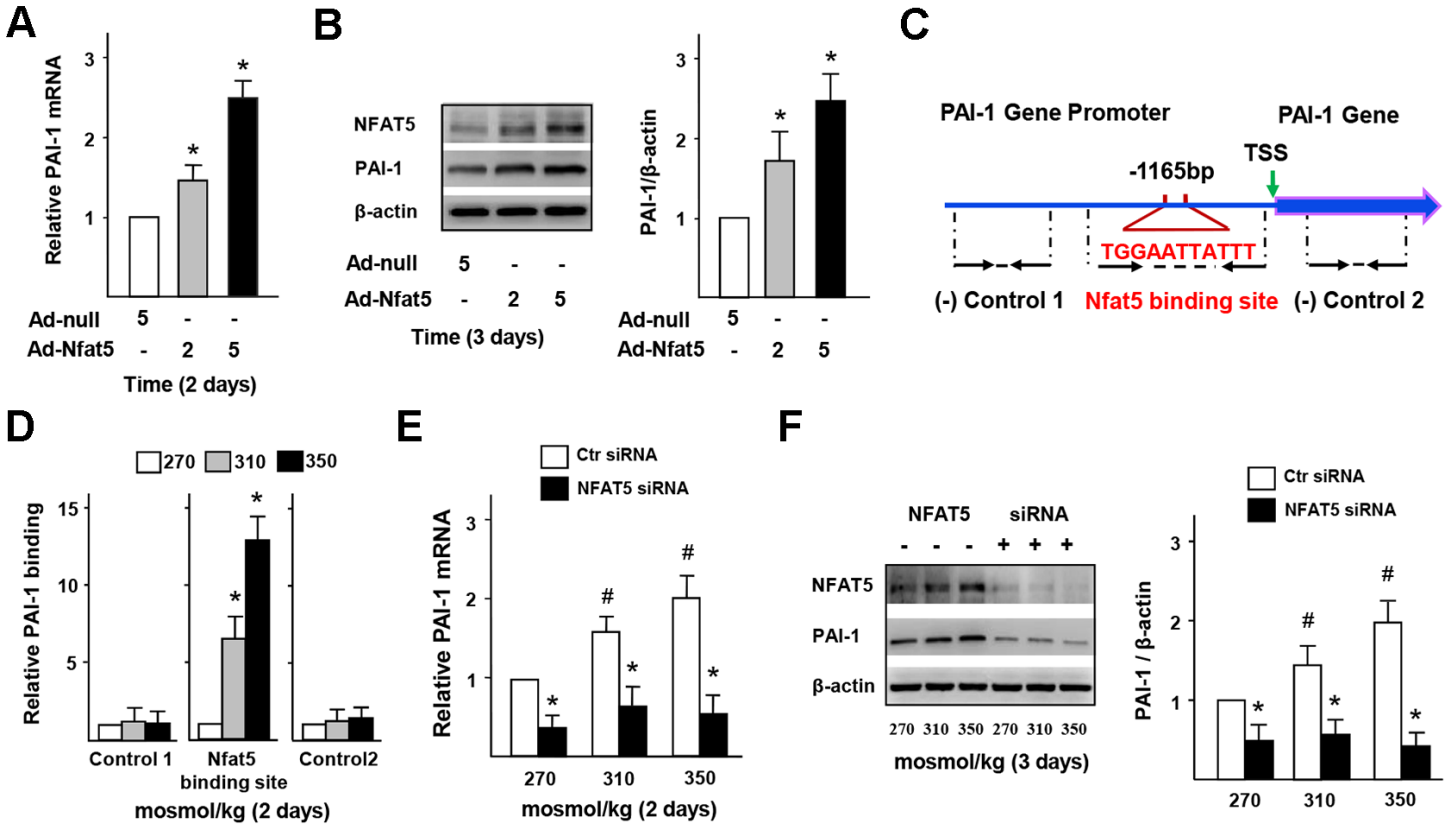
**NFAT5 directly regulates PAI-1 transcription in ECs.** (**A**, **B**) mRNA and protein expression of PAI-1 in HUVECs that treated by Ad-null or Ad-NFAT5. (**C**, **D**) High-salt increases binding of NFAT5 to PAI-1 promoter. Diagram showing the region of the NFAT5 binding site upstream of the transcription start site (TSS) of PAI-1, and the regions that were used to analyze NFAT5 binding by ChIP. ChIP results were relative to 270 mosmol/kg. (**E**, **F**) mRNA and protein expression of PAI-1 in HUVECs that transfected with Ctr siRNA or NFAT5 siRNA under high-salt condition. All data were presented as mean ± SEM, N≥3. *p < 0.05 versus control group.

The results of RT-PCR and western blot (Figure 7A, 7B) presented that the gene and protein expression of PAI-1 in Ad-NFAT5 group was significantly higher than that in the control group. In order to further explore the relationship between NFAT5 expression and PAI-1 transcription, we tested whether NFAT5 could effectively bind to the specific site in PAI-1 promoter. Through bioinformatics analysis, a target osmotic response element (ORE, NFAT5 binding site, TGGAATTATTT) was successfully selected near the PAI-1 promoter, and further verification of the CHIP results was obtained (Figure 7C, 7D and Supplementary Figure 6). The binding between NFAT5 and PAI-1 gene enhanced gradually with increasing salt concentration (Figure 7D), but decreased significantly under NFAT5 knockdown (Supplementary Figure 6). Figure 7E, 7F further showed that the gene and protein expression of PAI-1 was significantly decreased after knocking down NFAT5. ELISA results (Supplementary Figure 4B) also demonstrated a significant decrease in PAI-1 secretion of the NFAT5 siRNA-treated group. Together, these results indicated that NFAT5 could bind directly to PAI-1 promoter and increase PAI-1 secretion, thus promoting blood fibrinolysis and inflammatory cells infiltration in vascular under HS stimulation.

## DISCUSSION

**Figure 8:**
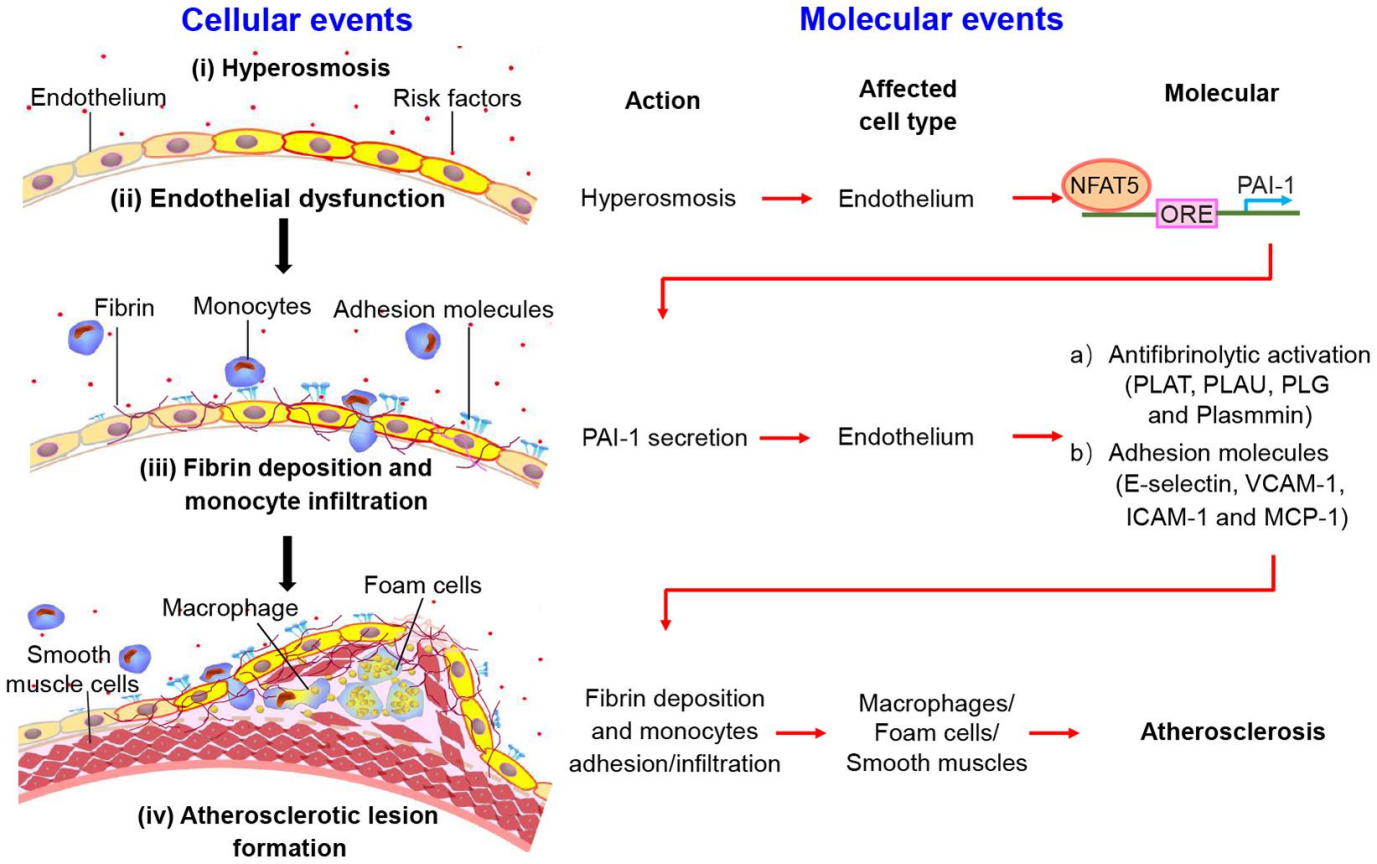
**The schematic diagram shows the process of fibrin deposition, macrophage infiltration and atherosclerosis formation.** Stage I: Hypertonicity → NFAT5-dependent PAI-1 gene transcription → PAI-1 secretion. Stage II: PAI-1 secretion → Antifibrinolytic activation/adhesive molecules → Fibrin deposition/monocytes adhesion and infiltration. Stage III: Endothelial dysfunction leads to fibrin deposition, macrophage-driven foam cells and phenotype conversion of smooth muscle cells, contributing to the formation of atherosclerotic plaque.

High level of serum sodium has been shown to cause endothelial dysfunction and initiate the development of atherosclerotic lesion. A key step at the early development of AS is fibrin deposition and macrophages infiltration in ECs. However, the cellular and molecular mechanisms by which HS mediates the fibrinolytic activation and adhesive property in ECs at the transcription level remain elusive. In this study, we identified that HS had great potential to promote the abundance and translocation of NFAT5, which activates PAI-1-mediated the antifibrinolytic activation and monocytes adhesion/transmigration in ECs, leading to accelerated fibrin deposition and macrophages infiltration in the artery wall, and eventually promoting the development of atherosclerotic lesions. Notably, we further validated that NFAT5 could significantly accelerate endothelial dysfunction by binding to the PAI-1 promoter and activating PAI-1 expression under HS conditions (Figure 8).

It has reported that transcription factors, such as Nur 77, EIK-1, and SP1, promote the PAI-1 gene transcription by binding to its promoter in ECs or other cells by various stimuli [28–30]. Here, our results suggested that the HS-elevated NFAT5 could increase the PAI-1 transcription by binding to its promoter, resulting in the dysfunction of the fibrinolysis and monocytes-adhesion/transmigration in ECs. The different molecular mechanisms of endothelial dysfunction induced by various risk factors via PAI-1 during AS are consistent with the “response-to-injury” hypothesis, which suggests that endothelial dysfunction precedes AS. As a response to hypertonic stress or other risk factors, some chronic injurious changes occur in ECs and initiate the formation of AS when normal functional thresholds are broken.

Based on previous researches and the results in this study, we concluded that HS can promote AS through NFAT5 - PAI - fibrin deposition/adhesion molecules - innate immunity - AS (Figure 8). Our results successfully demonstrated that the high expression of PAI-1 in ECs required an increase in the expression and translocation of NFAT5. The expression of PAI-1 is the key in converting plasminogen to active plasmin and elevating the levels of adhesive molecules in ECs under high-salt conditions, respectively. PAI-1 is a major mediator in the function of endothelial fibrinolysis and macrophages-infiltration in cardiovascular diseases. The elevated PAI-1 initiates ECs dysfunction and enhances AS susceptibility [31, 32]. Previous studies had demonstrated that HS could activate the expression of PAI-1 and adhesive molecules in endothelium [17, 18, 33]. The elevated PAI-1 was found to promote leukocytes infiltration and tissue injury after post ischemic [15, 34, 35]. Here, we also found that HS significantly promoted the gene and protein expression of PAI-1, impaired the plasmin-dependent dissolution of fibrin, and elevated the expression of adhesion molecules in ECs, resulting in the increased fibrin deposition and macrophages infiltration in the artery wall. These results further confirmed that endothelial dysfunction induced by high-salt intake can initiate the formation of AS. Importantly, this suggested that ECs established a dysfunctional threshold in response to risk factors for the generation of AS at the transcription level.

Growing evidence suggested that excessive PAI-1 and coagulators are generated by ECs in acute sepsis, reducing the fibrin degradation in blood which activates blood coagulation, and eventually lead to enhanced microvascular thrombosis [36, 37]. Previous study showed that high-plasma sodium by dehydration further control the production of vWF which enhances blood coagulation through the activation of NFAT5 [9]. Our study showed that HS intake activates NFAT5 to elevate PAI-1 secretion in ECs, which promotes fibrin deposition and macrophages infiltration into artery wall of mice, suggesting that NFAT5 is a key regulator of endothelial dysfunction in reducing AS. However, there is a contradictory idea on which the fibrin deposition promotes AS or AS causes the fibrin deposition. Interestingly, our studies showed there is an elevated fibrin deposition in the subendothelial layer of aortas in ApoE^-/-^ mice with HS intake. Conversely, there is a decreased expression of plasminogen in ECs under HS intake condition or NFAT5 overexpression, suggesting a low expression and activation of plasminogen in ECs may promote the early fibrin deposition in the aortas. Importantly, this indicates the early fibrin deposition may direct AS formation.

In the present, we demonstrated that an elevated expression and nuclear translocation of NFAT5 controls PAI-1 expression and its downstream pathway in the AA of the ApoE^-/-^ mice with HS intake. The important question is that how HS mediates NFAT5 activity. Biomechanical factors (*e.g.,* arterial wall stress) were found to stimulate the expression and translocation of NFAT5 through activating the c-Jun N-terminal kinase pathway [21], suggesting that disturbed-flow shear stress-regulated pathways may contribute to NFAT5 activation. Our results also showed that NFAT5 activation in the AA of aortas higher than that of the TA. In addition, many positive and negative regulators (e.g., p38 MAPK, PKA, and Fyn [38, 39]) have been identified in regulating NFAT5 activity. Thus, HS may chronically increase the blood pressure, resulting in the enlarged atherogenic areas by disturbed-flow shear stress and elevated arterial wall stress, which synergize with these regulators to activate NFAT5 expression and nuclear translocation. Our study also showed that transcription factor NFAT5 directly binds to PAI-1 promoters to promote the fibrinolytic activation and macrophages adhesion/infiltration in ECs, resulting in the accelerated AS.

In conclusion, our findings suggested that hypertonic stress, similar to other atherogenic risk factors, such as hypertension, hyperlipidemia, and disturbed flow, promotes PAI-1 expression via NFAT5 in ECs, thus initiating endothelial dysfunction. It promotes fibrin deposition and macrophages infiltration in the artery wall, and leads to the formation of AS. Notably, HS-stimulated NFAT5 activates the transcription activity of PAI-1 by binding to its promoter and activates the dysfunction of endothelial fibrinolysis and monocytes-adhesion/infiltration. The findings identified in this study provide a novel mechanism of atherosclerotic lesions formation, and suggest that ECs are the direct target cells of cardiovascular risk factors that initiate AS. The novel insights into the pathogenesis of AS have clear clinical applications. As the prevalence of severe cardiovascular diseases all over the world, maintaining the tissues below a dysfunctional endowed threshold that contribute to pathological changes would go far towards keeping healthy.

## MATERIALS AND METHODS

### Cell culture and animals

Human umbilical vein endothelial cells (HUVECs) were cultured with the endothelial growth medium. This medium was prepared by blending the following supplements, 2.5 ng/mL Thymide (Solarbio, Beijing, China), 0.108 mg/mL Heparin sodium (Solarbio, Beijing, China), 3.2 ng/mL β-Endothelial Cell Growth Factor (β-ECGF, Sigma, Billerica, MA) and 10% Fetal Bovine Serum (FBS, Biological Industries, Kibbutz Beit Haemek, Israel) with M199 medium (HyClone, South Logan, UT). Osmolality in normal endothelial growth medium was around 270 mosmol/kg, and then gradually elevated by adding NaCl (Sigma, Billerica, MA). The HUVECs at passages 2 - 6 were used in these experiments. Human THP-1 monocytes were maintained in RPMI 1640 medium (HyClone, South Logan, UT) and split 1:2 - 1:3 every 4 - 5 days at a density of three million per mL culture.

Local government authorities approved these animal experiments, according to internationally accepted laws. The apolipoprotein E-deficient (ApoE^-/-^) mice (8-week old) were purchased from Daping Hospital (Chongqing, China), and fed for another two weeks to adapt to the environment. We randomly divided male ApoE^-/-^ mice into normal-salt chow (0.8% NaCl) and high-salt chow (8% NaCl) groups.

### RT-PCR

Total RNA was isolated from HUVECs (culturing for 2 days) and used to synthesize cDNA. After that, RT-PCR measurements were performed with SYBR Green supermix (Bio-Rad, Hercules, CA). The target genes were finally normalized to β-actin.

After one month of feeding, the aortas of ApoE^-/-^ mice were separated and minced in liquid nitrogen. Then, the ground samples were transferred into tubes that contained RNA lysate buffer. The remaining steps referred to the procedures described above for cells. Supplementary Table 1 in supplementary showed the sequences of the primers used in these experiments.

### Western blot

Briefly, cells at three days were lysed in the RIPA buffer to collect the total proteins. Then samples were separated using SDS-PAGE electrophoretic gels (10%). After electrotransferring onto PVDF membranes, the blots were treated with different primary antibodies [NFAT5 (Abcam, Cambridge, MA), PAI-1 (Abcam, Cambridge, MA), PLAT (Proteintech, Chicago, IL), PLAU (Proteintech, Chicago, IL), PLG (Proteintech, Chicago, IL) or β-actin (Abcam, Cambridge, MA)] and horseradish peroxidase-conjugated secondary antibodies (ZSGB-BIO, Beijing, China). Protein bands were finally treated with ECL substrate solution and exposed to blue X-ray film to obtain the images.

### siRNA transfection

Cells were treated with 50 nM of NFAT5/PAI-1 or control siRNA for six hours, and then stimulated by different hypertonic medium for two or three days. After that, HUVECs were lysed, and target genes or proteins were analyzed as described above.

### Adenovirus infection

The logarithmically growing HUVECs were cotransfected with recombinant adenovirus encoding NFAT5 (Ad-NFAT5) or adenovirus null (Ad-null) (Cyagen, Guangzhou, China). After two or three days for infection, cells were harvested, and target genes or proteins were measured as described above.

### Chromatin immunoprecipitation (ChIP) assay

HUVECs were cultured in the gradient hypertonic medium with or without NFAT5 siRNA for two days. Then an Enzymatic CHIP Kit (Cell Signaling, Danvers, MA) was used to performed immunoprecipitation as recommended by the manufacturers’ protocols. Three specific primers were selected near the initiation site of PAI-1 gene. Among them, one sequence overlapped the NFAT5 binding site, while the others were not and served as negative controls, respectively.

### Immunofluorescence staining and En face immunostaining

HUVECs were exposed to different hypertonic medium for three days and then fixed with 4% (v/v) buffered paraformaldehyde (Solarbio, Beijing, China). After treated with 0.2% (v/v) Triton X-100 (Solarbio, Beijing, China), HUVECs were immersed overnight in the solution of PAI-1 primary antibody. Alexa Fluor 488 nm conjugated goat anti-rabbit secondary antibodies (Beyotime, Jiangsu, China) was used to combine with primary antibodies. After washing, HUVECs were stained with DAPI (Beyotime, Jiangsu, China) and pictures were taken using the microscope (Leica, Germany).

The aortas isolated from mice were cleaned, incised longitudinally, and fixed on the foam board. Then the ECs in endovascular layer were blocked and incubated with primary/secondary antibodies as before. All images were visualized using a confocal microscopy.

### Immunohistochemistry

Anesthetized mice were perfused by the ice-cold PBS and 4% paraformaldehyde. Kidney, aortas, and livers were separated and embedded in paraffin. 5 μm sections were prepared by microtome. Afterward, kidney sections were treated with rabbit PAI-1/CD31 primary (Cell Signaling, Danvers, MA) and HRP-conjugated secondary antibodies. Brown color could appear under the effect of the DAB substrate kit (Cell Signaling, Danvers, MA). At last, hematoxylin was used to counterstain cell nucleus. Likewise, aortas sections were stained using F4/80 (Cell Signaling, Danvers, MA) and fibrinogen antibodies (Abcam, Cambridge, MA); liver sections were treated with fibrinogen antibodies as mentioned above. Images were visualized using a microscope.

### Oil red O staining

After feeding for 12 weeks, mice were anesthetized and killed. The aortas were collected, dissected longitudinally, fixed on the foam board with pins, and stained using Oil Red O solution. After being rinsed, the vessels were captured under the anatomical lens (Guangxi, China). The extent of disease was measured as a percentage of the plaque area in the overall surface area of the vessel.

### Statistical analysis

Data were analyzed with Student’s t-test and one-way ANOVA, and presented as mean ± SEM. P < 0.05 was considered significant.

## Supplementary Material

Supplementary Materials and Methods

Supplementary Figures

Supplementary Table 1
