# Supplementary material for: NFAT5 directs hyperosmotic stress-induced fibrin deposition and macrophage infiltration via PAI-1 in endothelium
Source: Aging (Albany NY). 2020 Dec 19;13(3):3661–79. doi: 10.18632/aging.202330 (PMC7906158; doi:10.18632/aging.202330)
Supplement: Supplementary Materials and Methods [file aging-13-202330-s001.pdf]

SUPPLEMENTARY MATERIALS AND METHODS

Elisa

HUVECs were cultured in serum-reduced (about 2%) media and transfected with NFAT5 or control siRNA before stressed by NaCl. After three days, we collected the culture supernatants. Thereafter, the secretions of PAI-1 and plasmin were assessed by using the Human PAI-1 ELISA Kit (Abcam, Cambridge, MA) and Human plasmin ELISA Kit (Jianglaibio, Shanghai, China), respectively. Furthermore, total proteins in ECs were extracted and measured as described above. The final PAI-1 levels were calculated from divided PAI-1 secretions by respective total protein. Afterward, the rate of PAI-1 levels in the samples were normalized to the control.

After feeding for 4 weeks, the blood of $ApoE^{-/-}$ mice were collected and protected by 50 uL of heparin sodium. Then the levels of PAI-1, plasmin, and D-Dimer in the platelet-free plasma were determined using Mouse PAI-1 ELISA Kit (Abcam, Cambridge, MA), Mouse plasmin ELISA Kit (Jianglaibio, Shanghai, China) and Mouse D2D (D-Dimer) ELISA Kit (Elabscience, Wuhan, China), according to the steps provided by the instructions.

Monocyte-HUVEC adhesion assay

Planted HUVECs were treated with PAI-1 or control siRNA followed by an hypertonic stimulation for three days. THP-1 were washed gently and labeled with 10 µM Calcein (Invitrogen, Carlsbad, CA) in PBS. An equal amount of monocytes were added into 6-wells plates and incubated with the ECs for half an hour at 37° C in the dark. Wash gently and take images with a microscope (Leica, Germany).

Transendothelial migration assay

In vitro, the transendothelial migration of monocytes experiments were performed using a transwell chamber (5 µm pore size polycarbonate membrane, Corning, Bedford, MA). Briefly, after treated by PAI-1 or control siRNA, HUVECs were stimulated in hypertonic medium for two days. Then the treated HUVECs were digested and seeded evenly in the upper chamber. Adequate cells were used to ensure that the cells were covered the bottom of the upper chamber after 8 h. Then Dil-labeled THP-1 cells were suspended in serum-free M199 medium contained different sodium concentration and seeded onto the upper chamber. Meanwhile, the M199 medium supplemented with 5% FBS was filled into the lower chamber. After another 24 h, the THP-1 cells that migrated through the ECs were pictured under a microscope, and the numbers were counted.

Serum sodium and cholesterol concentration

After feeding for 4 weeks, serum was collected from anesthetized mice. The levels of sodium and cholesterol in the mice serum were finally determined by Chongqing University Hospital.

Supplementary Figures

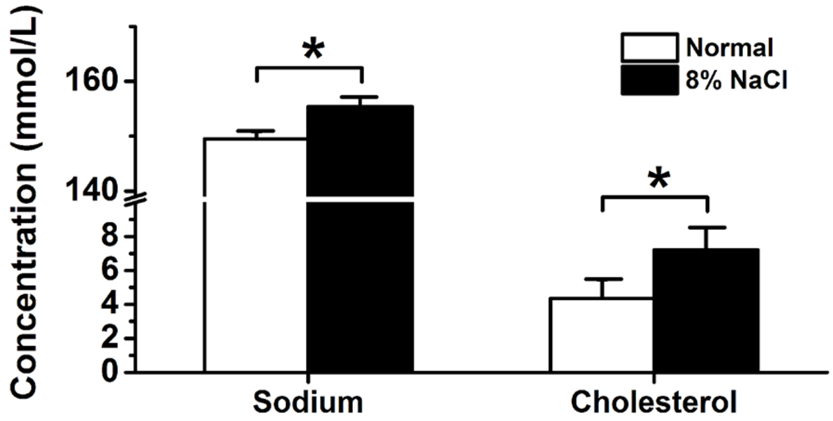

Supplementary Figure 1. The concentration of serum sodium and cholesterol in ApoE$^{-/-}$ mice feeding for 4 weeks. All data were presented as mean ± SEM, N ≥ 3. *p < 0.05 versus control group.

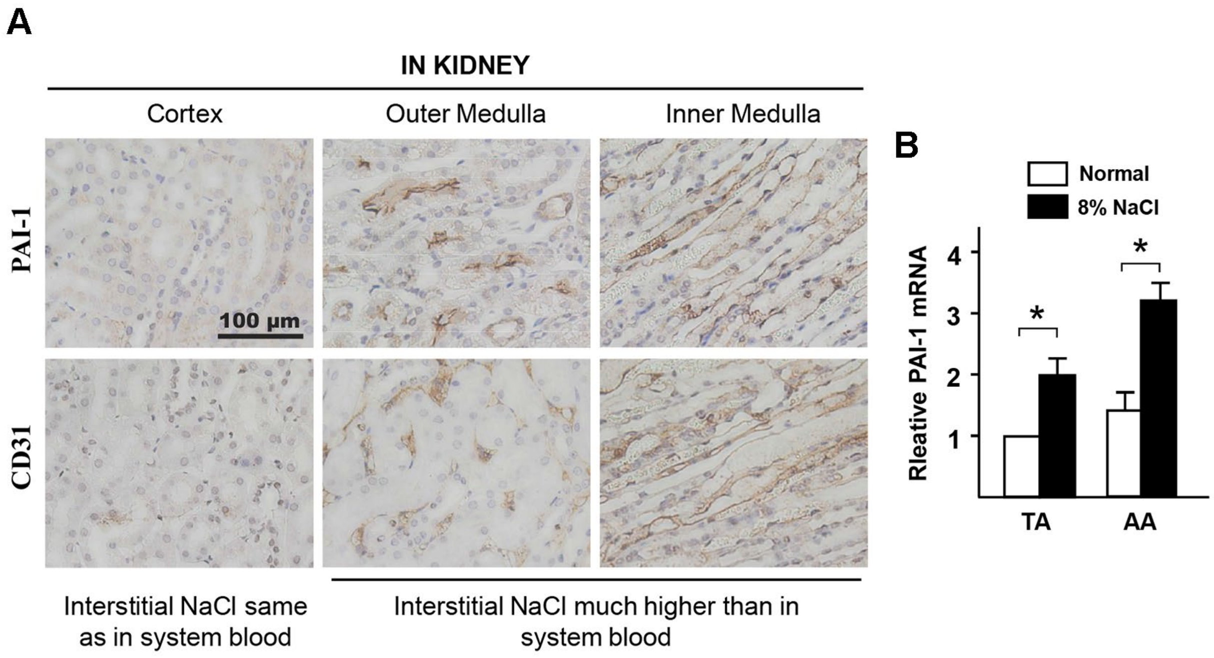

Supplementary Figure 2. High-salt increases PAI-1 expression in ECs. (**A**) Representative immunohistochemistry staining for PAI-1 in the kidney of mice feeding for 4 weeks. Nuclei, hematoxylin staining. (**B**) mRNA expression of PAI-1 in TA and AA regions of ApoE$^{-/-}$ mice in normal and high salt groups after 4 weeks feeding. All data were presented as mean ± SEM, N ≥ 3. *p < 0.05 versus control group.

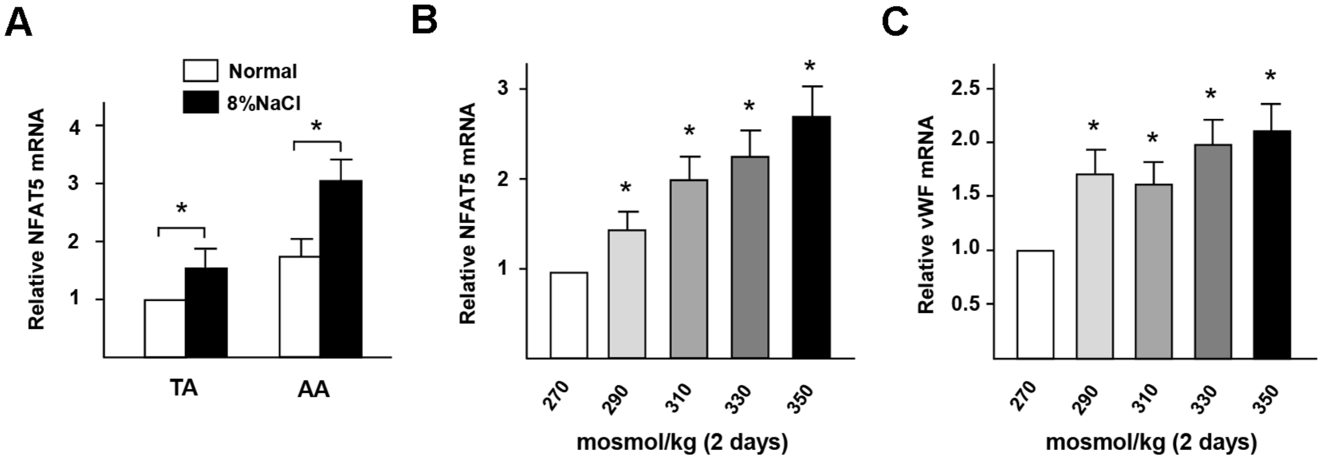

Supplementary Figure 3. High-salt increases the mRNA expression of NFAT5 and vWF in ECs. (**A**) mRNA expression of NFAT5 in TA and AA regions of $ApoE^{-/-}$ mice in normal and high salt groups after 4 weeks feeding. (**B**, **C**) mRNA expression of NFAT5 and vWF in HUVECs that exposed to different hyper-osmotic media for two days. All data were presented as mean ± SEM, N ≥ 3. *p < 0.05 versus control group.

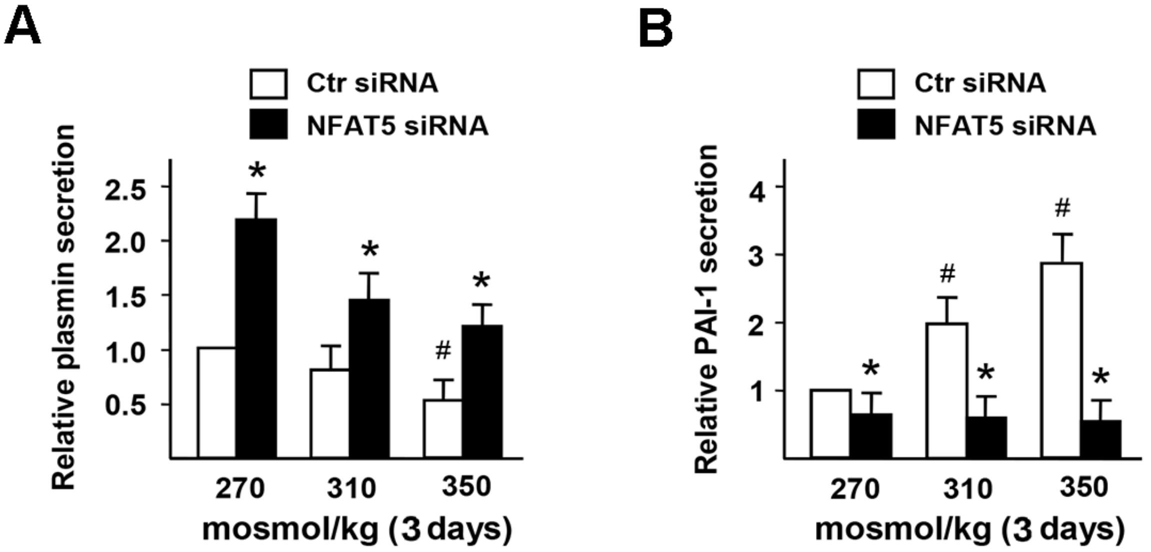

Supplementary Figure 4. High-salt induces the dysfunction of PAI-1-dependent fibrinolysis in ECs via NFAT5. (**A**) The secretion of active plasmin in HUVECs that transfected with Ctr siRNA or NFAT5 siRNA under high-salt condition. (**B**) The secretion of PAI-1 protein in HUVECs that transfected with Ctr siRNA or NFAT5 siRNA under high-salt condition. All data were presented as mean ± SEM, N ≥ 3. *p < 0.05 versus control group.

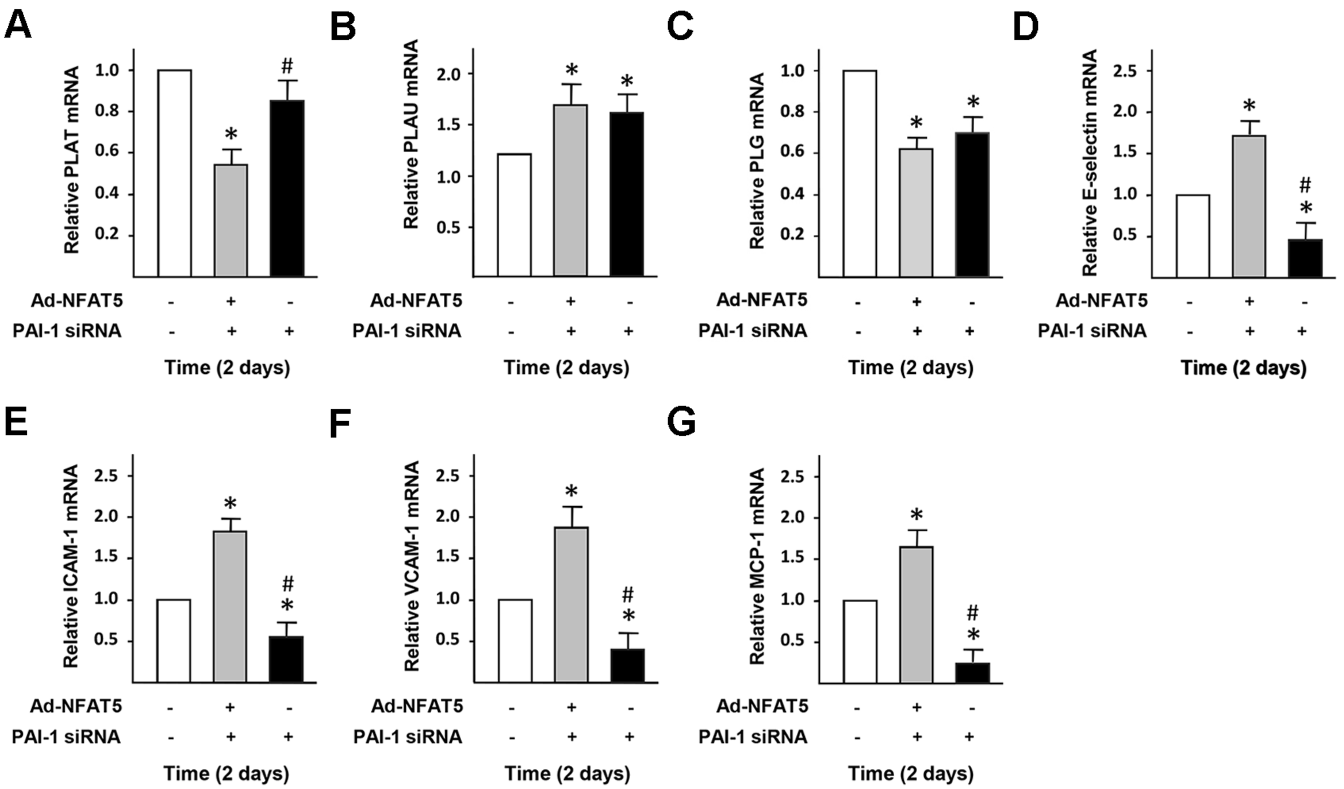

Supplementary Figure 5. The effect of PAI-1 knockdown on the dysfunction of endothelial fibrinolysis and monocytes adhesion in NFAT5-overexpressing cells. (**A**–**C**) mRNA expression of fibrinolysis genes (PLAT, PLAU and PLG) in ECs that transfected by Adenovirus-NFAT5 and/or PAI-1 siRNA. (**D**–**G**) mRNA expression of adhesive molecules (E-selectin, ICAM-1, VCAM-1, and MCP-1) in ECs that transfected by Adenovirus-NFAT5 and/or PAI-1 siRNA. All data were presented as mean ± SEM, N ≥ 3. *p < 0.05 versus control group.

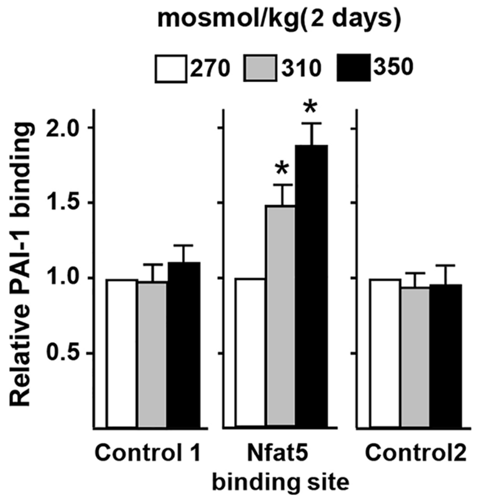

Supplementary Figure 6. The binding of NFAT5 to PAI-1 promoter in HUVECs that transfected with NFAT5 siRNA under high-salt condition. All data were presented as mean ± SEM, N ≥ 3. *p < 0.05 versus control group.

Supplementary Table

Supplementary Table 1. The primers sequences for RT-qPCR in this study.

Target genes	Primer sequences
Human NFAT5	**Forward:** 5'- ACAGTAAAGCTGGAAGGCCA -3'
	**Reverse:** 5'- TTGCTAGGATCAAGGCCGAC -3'
Human PAI-1	**Forward:** 5'- ACCGCAACGTGGTTTTCTCA'
	**Reverse:** 5'- TTGAATCCCATAGCTGCTTGAAT -3'
Human PLG	**Forward:** 5'-CAGGGGGCTTCACTGTTCAG -3'
	**Reverse:** 5'- GCCATTATCACACATTGTTGCTC -3'
Human β-actin	**Forward:** 5'- CGAGCGCGGCTACAGCTT -3'
	**Reverse:** 5' - TCCTTAATGTCACGCACGATTT -3'
Human PLAT	**Forward:** 5'- TGCTGTGAAATAGATACCAGGGC -3'
	**Reverse:** 5'- TGAGTCTCGATCTGGGTTTCTG -3'
Human PLAU	**Forward:** 5'- GCTTGTCCAAGAGTGCATGGT -3'
	**Reverse:** 5'- CAGGGCTGGTTCTCGATGG -3'
Human VCAM-1	**Forward:** 5'- GGGAAGATGGTCGTGATCCTT -3'
	**Reverse:** 5'- TCTGGGGTGGTCTCGATTTTA -3'
Human ICAM-1	**Forward:** 5'- ATGCCCAGACATCTGTGTCC -3'
	**Reverse:** 5'- GGGGTCTCTATGCCCAACAA -3'
Human MCP-1	**Forward:** 5'- AGCATGAAAGTCTCTGCCGCCCTTCTG -3'
	**Reverse:** 5'- ATTACTTAAGGCATAATGTTTCACA -3
Human E-sele	**Forward:** 5'- AGAGTGGAGCCTGGTCTTACA -3'
	**Reverse:** 5'- CCTTTGCTGACAATAAGCACTGG -3'
Mouse NFAT5	**Forward:** 5'- ATCGCCCAAGTCCCTGTACT -3'
	**Reverse:** 5'- GCTTGTCTGACTCATTGATGCTA -3'
Mouse PAI-1	**Forward:** 5'- TCTGGGAAAGGGTTCACTTTACC -3'
	**Reverse:** 5'- GACACGCCATAGGGAGAGAAG -3'
Mouse PLAT	**Forward:** 5'- TGACCAGGGAATACATGGGAG -3'
	**Reverse:** 5'- CTGAGTGGCATTGTACCAGGC -3'
Mouse PLAU	**Forward:** 5'- GGAGGTGTATGCGTGTCCTAC -3'
	**Reverse:** 5'- CCATGATAGCAGGTTTTTGATGC -3'
Mouse PLG	**Forward:** 5'- TGCAGTGGAGAAAAGTATGAGGG -3'
	**Reverse:**5'- AGGGATGTATCCATGAGCATGT -3'
Mouse VCAM-1	**Forward:** 5'- TTGGGAGCCTCAACGGTACT -3'
	**Reverse:** 5'- GCAATCGTTTTGTATTCAGGGGA -3'
Mouse ICAM-1	**Forward:** 5'- GTGATGCTCAGGTATCCATCCA -3'
	**Reverse:** 5'- CACAGTTCTCAAAGCACAGCG -3'
Mouse MCP-1	**Forward:** 5'- TTAAAAACCTGGATCGGAACCAA -3'
	**Reverse:** 5'- GCATTAGCTTCAGATTTACGGGT -3'
Mouse E-selectin	**Forward:** 5'- CCAATCTGAAACATTCACCGAGT -3'
	**Reverse:** 5'- GAGTCTTTGGTTCGTTGGATGTA -3'
Mouse β-actin	**Forward:** 5'- GTGACGTTGACATCCGTAAAGA -3'
	**Reverse:** 5' - GCCGGACTCATCGTACTCC -3'
