# Supplementary material for: NFAT5 directs hyperosmotic stress-induced fibrin deposition and macrophage infiltration via PAI-1 in endothelium
Source: Aging (Albany NY). 2020 Dec 19;13(3):3661–79. doi: 10.18632/aging.202330 (PMC7906158; doi:10.18632/aging.202330)
Supplement: Supplementary Figures [file aging-13-202330-s002.pdf]

SUPPLEMENTARY FIGURES

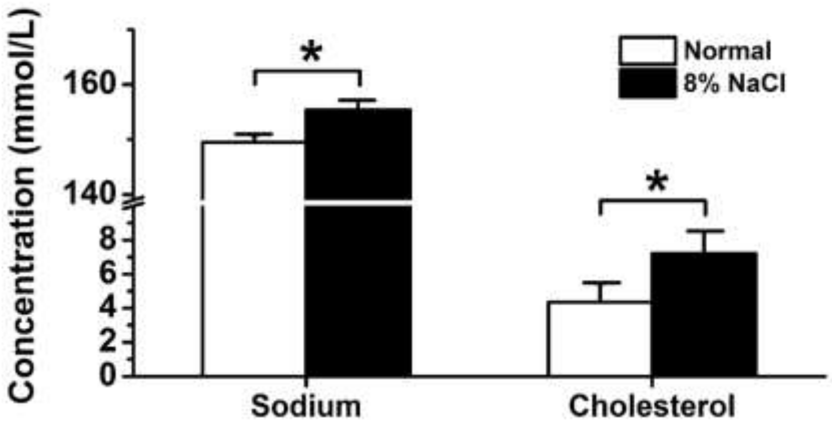

Supplementary Figure 1. The concentration of serum sodium and cholesterol in ApoE$^{-/-}$ mice feeding for 4 weeks. All data were presented as mean ± SEM, N ≥ 3. *p < 0.05 versus control group.

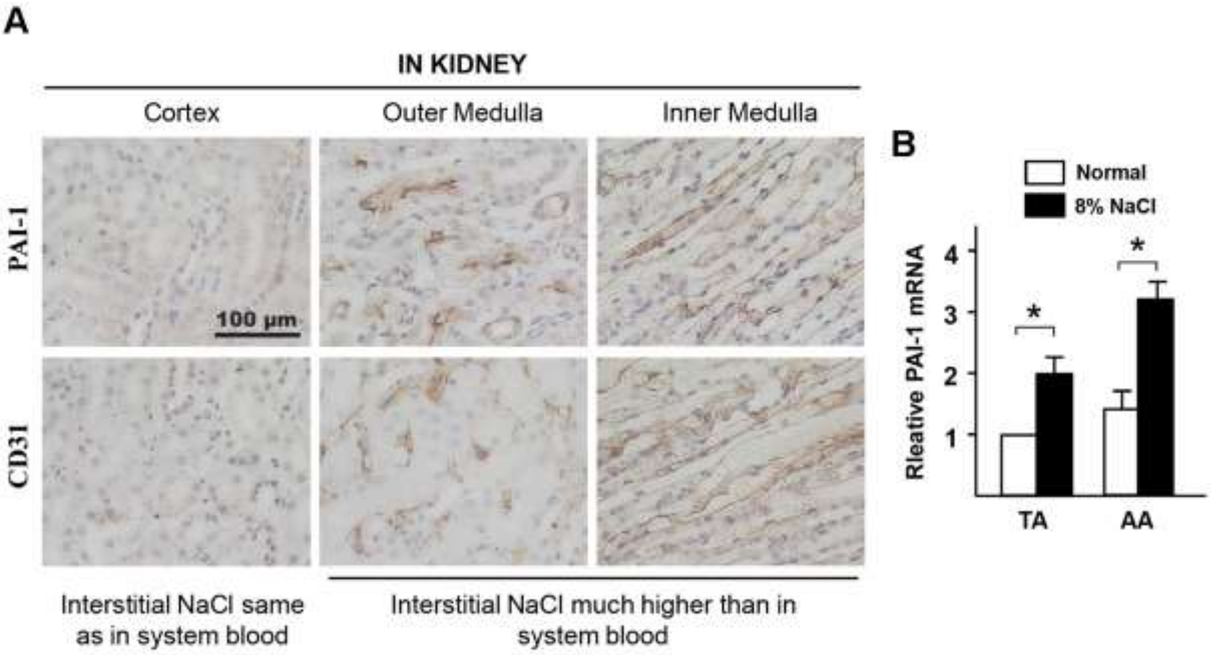

Supplementary Figure 2. High-salt increases PAI-1 expression in ECs. (**A**) Representative immunohistochemistry staining for PAI-1 in the kidney of mice feeding for 4 weeks. Nuclei, hematoxylin staining. (**B**) mRNA expression of PAI-1 in TA and AA regions of ApoE$^{-/-}$ mice in normal and high salt groups after 4 weeks feeding. All data were presented as mean ± SEM, N ≥ 3. *p < 0.05 versus control group.

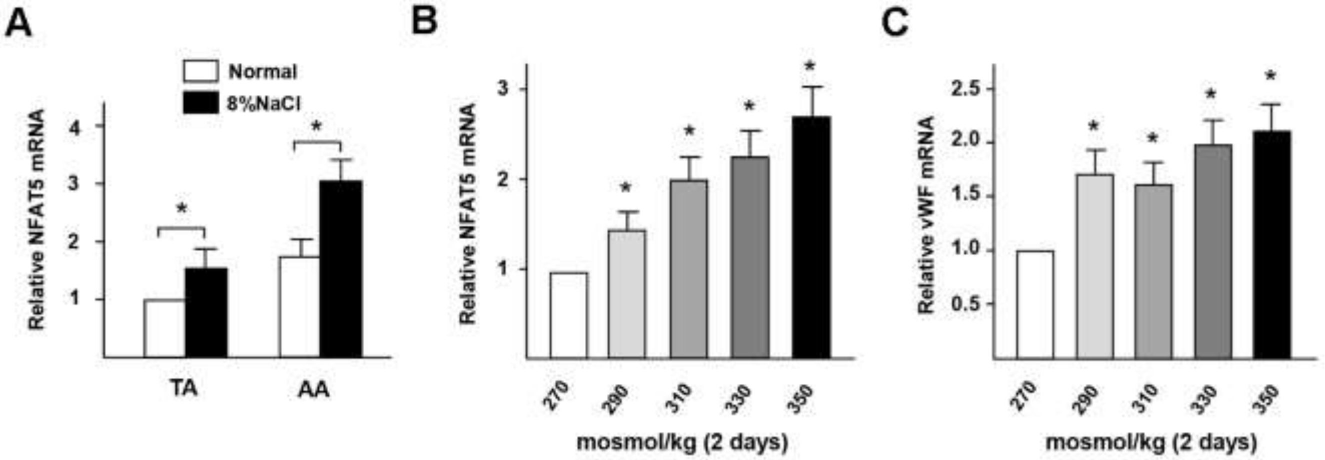

Supplementary Figure 3. High-salt increases the mRNA expression of NFAT5 and vWF in ECs. (**A**) mRNA expression of NFAT5 in TA and AA regions of ApoE$^{-/-}$ mice in normal and high salt groups after 4 weeks feeding. (**B**, **C**) mRNA expression of NFAT5 and vWF in HUVECs that exposed to different hyper-osmotic media for two days. All data were presented as mean ± SEM, N ≥ 3. *p < 0.05 versus control group.

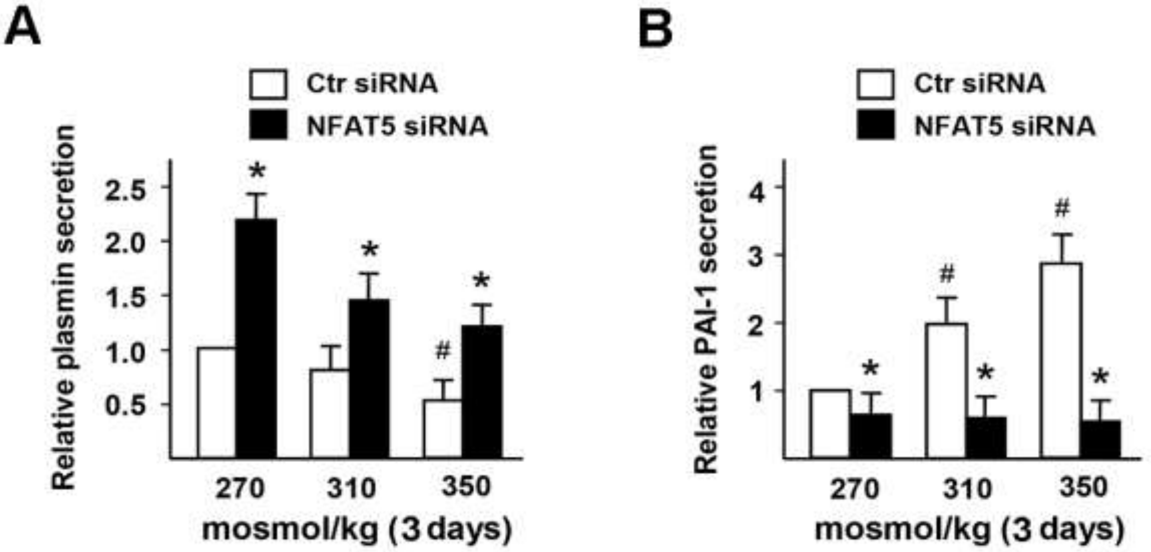

Supplementary Figure 4. High-salt induces the dysfunction of PAI-1-dependent fibrinolysis in ECs via NFAT5. (**A**) The secretion of active plasmin in HUVECs that transfected with Ctr siRNA or NFAT5 siRNA under high-salt condition. (**B**) The secretion of PAI-1 protein in HUVECs that transfected with Ctr siRNA or NFAT5 siRNA under high-salt condition. All data were presented as mean ± SEM, N ≥ 3. *p < 0.05 versus control group.

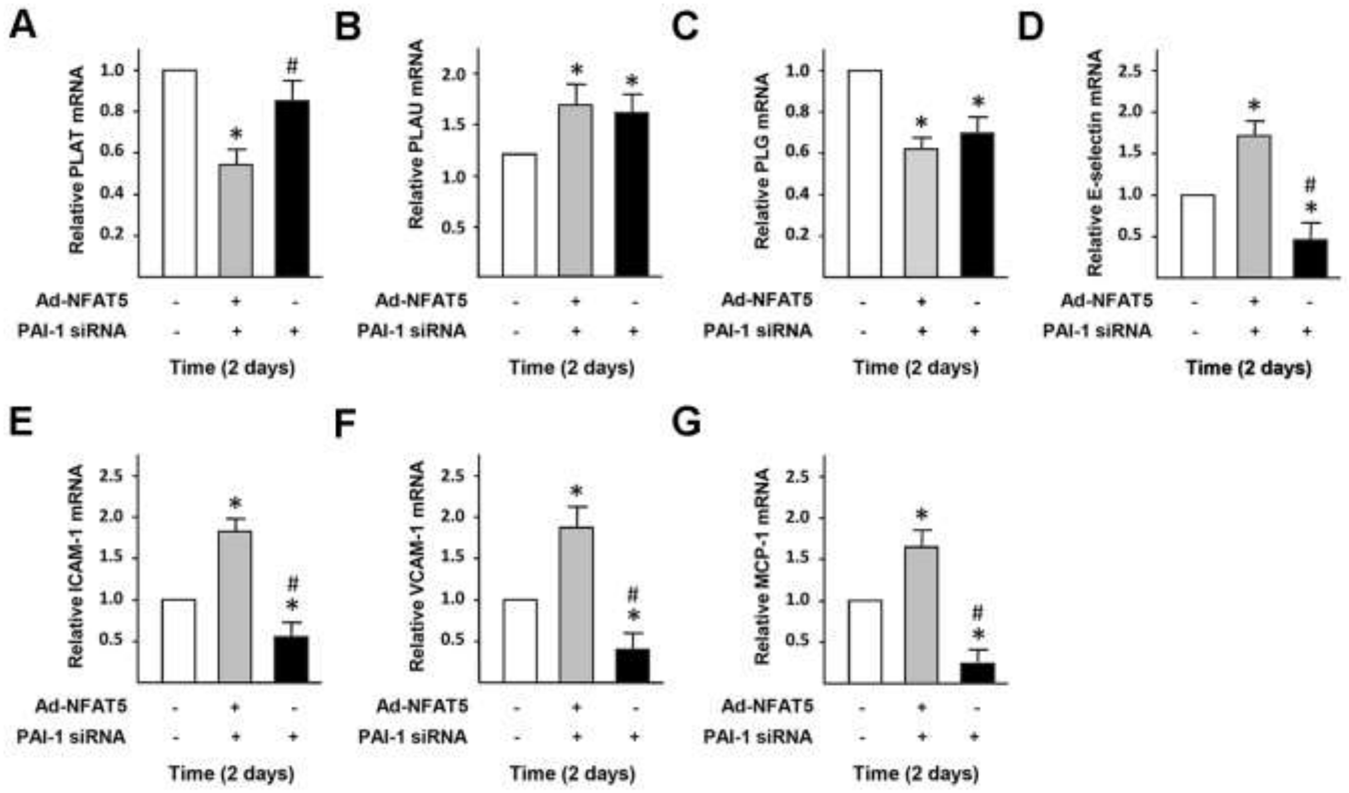

Supplementary Figure 5. The effect of PAI-1 knockdown on the dysfunction of endothelial fibrinolysis and monocytes adhesion in NFAT5-overexpressing cells. (**A**–**C**) mRNA expression of fibrinolysis genes (PLAT, PLAU and PLG) in ECs that transfected by Adenovirus-NFAT5 and/or PAI-1 siRNA. (**D**–**G**) mRNA expression of adhesive molecules (E-selectin, ICAM-1, VCAM-1, and MCP-1) in ECs that transfected by Adenovirus-NFAT5 and/or PAI-1 siRNA. All data were presented as mean ± SEM, N ≥ 3. *p < 0.05 versus control group.

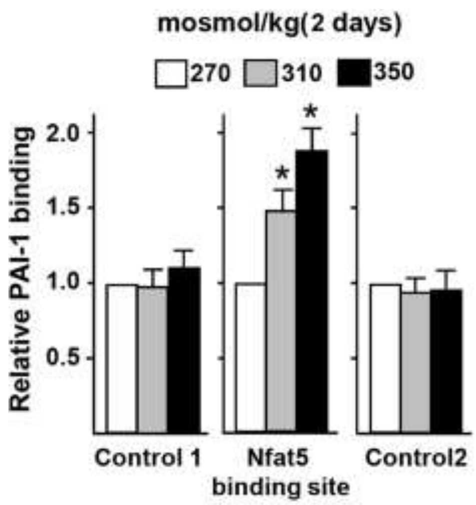

Supplementary Figure 6. The binding of NFAT5 to PAI-1 promoter in HUVECs that transfected with NFAT5 siRNA under high-salt condition. All data were presented as mean ± SEM, N ≥ 3. *p < 0.05 versus control group.
