# Supplementary material for: NFAT5 directs hyperosmotic stress-induced fibrin deposition and macrophage infiltration via PAI-1 in endothelium
Source: Aging (Albany NY). 2020 Dec 19;13(3):3661–79. doi: 10.18632/aging.202330 (PMC7906158; doi:10.18632/aging.202330)
Supplement: Supplementary Table 1 [file aging-13-202330-s003.pdf]

SUPPLEMENTARY TABLE

Supplementary Table 1. The primers sequences for RT-qPCR in this study.

Target genes	Primer sequences
Human NFAT5	**Forward:** 5'- ACAGTAAAGCTGGAAGGCCA -3'
	**Reverse:** 5'- TTGCTAGGATCAAGGCCGAC -3'
Human PAI-1	**Forward:** 5'- ACCGCAACGTGGTTTTCTCA'
	**Reverse:** 5'- TTGAATCCCATAGCTGCTTGAAT -3'
Human PLG	**Forward:** 5'-CAGGGGGCTTCACTGTTCAG -3'
	**Reverse:** 5'- GCCATTATCACACATTGTTGCTC -3'
Human β-actin	**Forward:** 5'- CGAGCGCGGCTACAGCTT -3'
	**Reverse:** 5' - TCCTTAATGTCACGCACGATTT -3'
Human PLAT	**Forward:** 5'- TGCTGTGAAATAGATACCAGGGC -3'
	**Reverse:** 5'- TGAGTCTCGATCTGGGTTTCTG -3'
Human PLAU	**Forward:** 5'- GCTTGTCCAAGAGTGCATGGT -3'
	**Reverse:** 5'- CAGGGCTGGTTCTCGATGG -3'
Human VCAM-1	**Forward:** 5'- GGGAAGATGGTCGTGATCCTT -3'
	**Reverse:** 5'- TCTGGGGTGGTCTCGATTTTA -3'
Human ICAM-1	**Forward:** 5'- ATGCCCAGACATCTGTGTCC -3'
	**Reverse:** 5'- GGGGTCTCTATGCCCAACAA -3'
Human MCP-1	**Forward:** 5'- AGCATGAAAGTCTCTGCCGCCCTTCTG -3'
	**Reverse:** 5'- ATTACTTAAGGCATAATGTTTCACA -3
Human E-sele	**Forward:** 5'- AGAGTGGAGCCTGGTCTTACA -3'
	**Reverse:** 5'- CCTTTGCTGACAATAAGCACTGG -3'
Mouse NFAT5	**Forward:** 5'- ATCGCCCAAGTCCCTGTACT -3'
	**Reverse:** 5'- GCTTGTCTGACTCATTGATGCTA -3'
Mouse PAI-1	**Forward:** 5'- TCTGGGAAAGGGTTCACTTTACC -3'
	**Reverse:** 5'- GACACGCCATAGGGAGAGAAG -3'
Mouse PLAT	**Forward:** 5'- TGACCAGGGAATACATGGGAG -3'
	**Reverse:** 5'- CTGAGTGGCATTGTACCAGGC -3'
Mouse PLAU	**Forward:** 5'- GGAGGTGTATGCGTGTCCTAC -3'
	**Reverse:** 5'- CCATGATAGCAGGTTTTTGATGC -3'
Mouse PLG	**Forward:** 5'- TGCAGTGGAGAAAAGTATGAGGG -3'
	**Reverse:**5'- AGGGATGTATCCATGAGCATGT -3'
Mouse VCAM-1	**Forward:** 5'- TTGGGAGCCTCAACGGTACT -3'
	**Reverse:** 5'- GCAATCGTTTTGTATTCAGGGGA -3'
Mouse ICAM-1	**Forward:** 5'- GTGATGCTCAGGTATCCATCCA -3'
	**Reverse:** 5'- CACAGTTCTCAAAGCACAGCG -3'
Mouse MCP-1	**Forward:** 5'- TTAAAAACCTGGATCGGAACCAA -3'
	**Reverse:** 5'- GCATTAGCTTCAGATTTACGGGT -3'
Mouse E-selectin	**Forward:** 5'- CCAATCTGAAACATTCACCGAGT -3'
	**Reverse:** 5'- GAGTCTTTGGTTCGTTGGATGTA -3'
Mouse β-actin	**Forward:** 5'- GTGACGTTGACATCCGTAAAGA -3'
	**Reverse:** 5' - GCCGGACTCATCGTACTCC -3'
